# Virtual reality (VR) to reduce anxiety in children in the plaster room: a study protocol for a randomised controlled trial

**DOI:** 10.1186/s13063-022-06488-2

**Published:** 2022-07-15

**Authors:** Lisa van der Water, Max A. Poppelaars, Iris Koenraadt-van Oost, Pieter Boele van Hensbroek, Christiaan J. A. van Bergen

**Affiliations:** 1 Department of Orthopaedic Surgery, Amphia Hospital, Breda, The Netherlands; 2 Foundation for Orthopaedic Research, Care & Education, Amphia Hospital, Breda, The Netherlands; 3 Department of Trauma Surgery, Amphia Hospital, Breda, The Netherlands

**Keywords:** Virtual reality, VR, Children, Anxiety, Plaster, Fracture, Distraction, Paediatric

## Abstract

**Background:**

Paediatric fractures are highly prevalent and are most often treated with plaster. The removal of plaster is often an anxious experience for children. Virtual reality (VR) has proven to effectively distract children and reduce their anxiety in other clinical settings. This study aims to investigate the effect of VR on the anxiety level of children with fractures that undergo plaster removal or replacement in the plaster room.

**Methods:**

This study is designed as a randomised controlled trial (RCT). The sample size is 270 patients, aged 5 to 17 years, with a fracture of the upper or lower extremity treated with plaster. The intervention group will be distracted with VR goggles and headphones during the replacement or removal of the plaster, whereas the control group will receive standard care. As a primary outcome, the level of anxiety will be measured with the Child Fear Scale (CFS). Secondary outcomes include anxiety reduction (difference between CFS after and CFS before plaster procedure) and Numeric Rating Scales (NRS) pain and satisfaction. Additionally, the children’s fastest heart rate during the procedure will be recorded. An unpaired samples *t*-test or a Mann-Whitney *U* test (depending on the data distribution) will be used to analyse the data.

**Discussion:**

When completed, this trial will provide evidence on the potential role of VR in children with fractures treated with plaster. The purpose is to increase the quality of healthcare by decreasing anxiety and possibly pain perception of children during a plaster procedure.

**Trial registration:**

Netherlands Trial Register NL9065. Registered on 27 November 2020

## Administrative information

Note: The numbers in curly brackets in this protocol refer to SPIRIT checklist item numbers. The order of the items has been modified to group similar items (see https://clicktime.symantec.com/3S6ewNUXrUE2dhpUYYBaKws6Gu?u=http%3A%2F%2Fwww.equator-network.org%2Freporting-guidelines%2Fspirit-2727-statement-defining-standard-protocol-items-for-clinical-trials%2F).

**Table:** 

Title {1}	Virtual reality (VR) to reduce anxiety in children in the plaster room: a study protocol for a monocenter randomised controlled trial
Trial registration {2a and 2b}.	Netherlands Trial Register, NL9065. Registered on 27 November 2020.
Protocol version {3}	Version 1, date: 17 September 2020 Version 2, date: 4 January 2021, Primary reason for new version: adjustments advised by the METC. Primary changes: Section 3 regarding the study design. Section 4.2 regarding the inclusion criteria. Section 4.3 regarding the exclusion criteria. Section 8.1.3 regarding descriptive data collected. Section 8.2 regarding the randomisation. Section 8.4 regarding the circumstances for withdrawal. Section 11.2 regarding the recruitment and consent procedure. Additional changes: language changes and minor changes based on the changes made in the sections above: summary and sections: 1, 2, 4.1, 4.4, 5.1, 8.1.1, 8.1.2, 8.3, 10.2, 10.3, 11.1, 11.3, 11.4, 11.5, 12.1, 12.6.
Funding {4}	The funding for this study is provided by the Amphia Hospital science fund (“Amphia Wetenschapsfonds”), Breda, The Netherlands. The equipment (Oculus Go, Oculus Quest 2 (VR goggles) and iPads) are provided by Vrienden van Amphia, Jack Rabbit Foundation, TopOpKids and Kiwanis.
Author details {5a}	CvB conceived the study. CvB and IvO initiated the study design, and PvH, MP and LvdW helped with the implementation. All authors contributed to the refinement of the study protocol and approved the final manuscript.
Name and contact information for the trial sponsor {5b}	Amphia Hospital, Molengracht 21 4818 CK Breda, The Netherlands Contact information: C.J.A. van Bergen (CvBergen@amphia.nl)
Role of sponsor {5c}	This funding source had no role in the design of this study and will not have any role during its execution, analyses, interpretation of the data or decision to submit results. There are no conflicts of interest.

## Introduction

### Background and rationale {6a}

Fractures occur frequently in children; the prevalence of paediatric fractures in The Netherlands is 40% for males and 28% for females between the ages of 6 and 16 [1]. The far majority of these children receive plaster treatment. The removal of plaster is often an anxious experience for children, particularly under the age of 13 [2, 3]. Methods to effectively reduce this anxiety may improve their hospital experience.

Different methods have been investigated to reduce anxiety in children in various clinical situations. Many of these methods have proven to be ineffective, such as using midazolam [4] and showing an instructional video ahead of the procedure [5]. However, a few methods were found that reduced the anxiety: watching videos on a smartphone or tablet during the procedure [6, 7] and using noise-reducing headphones during plaster removal [2]. Virtual reality (VR) has proven to reduce anxiety and pain perception of children in other clinical situations, such as blood draws, dental procedures, intravenous injections and treatment of burns [8–12]. The use of VR may be more effective in reducing anxiety and pain perception than a tablet [7]. A pilot study on VR in the plaster room has shown promising results [13].

Therefore, the aim of this study is to further investigate the anxiety-reducing effects of VR in a large group of children with fractures who undergo plaster replacement or removal. Our hypothesis is that the use of VR will reduce the anxiety of children with fractures in the plaster room and increase their satisfaction. We also hypothesise that younger children will benefit more than older children, based on experience with ear protection [3].

### Objectives {7}

The primary objective of this study is to evaluate the difference in the post-procedural anxiety level in children in the plaster room with or without distraction with use of VR. The secondary objectives of this study include anxiety reduction (difference between CFS scores pre- and post-procedural), level of pain experienced (using the numeric rating scale (NRS)), satisfaction scores (NRS) given by the child and the accompanying parent/guardian and the child’s fastest heart rate measured by a finger pulse oximeter during the procedure.

### Trial design {8}

The study is designed as a randomised controlled monocenter superiority trial with two parallel groups, with the children’s anxiety as the primary outcome. The randomisation will be stratified for age with a 1:1 allocation.

## Methods: participants, interventions and outcomes

### Study setting {9}

This study will be performed in the plaster room of the Amphia Hospital in Breda, The Netherlands.

### Eligibility criteria {10}

Patients and/or both legal guardians (depending on the child’s age) must provide written, informed consent before any study procedures occur (see Appendix 1–6 for the various informed consent forms).

#### Inclusion criteria

In order to be eligible to participate in this study, a subject must meet all of the following criteria:

1. Age 5–17 years.
2. At least one fractured bone in the upper or lower extremity.
3. Needs replacement or removal of plaster.
4. Children can only participate once in this study.

#### Exclusion criteria

A potential subject who meets any of the following criteria will be excluded from participation in this study:

1. Children who have already participated in this study at a previous plaster treatment
2. Children with known mental retardation, anxiety disorder, psychosis or epilepsy
3. Children with an extreme visual impairment (i.e. myopia > 8 dioptres or presbyopia > 5 dioptres)
4. Children and parents with an insufficient understanding of the Dutch or English language to give informed consent

### Who will take informed consent? {26a}

The informed consent will be obtained by the researcher. The children and parents will first be informed by posters and flyers in the emergency room. Subsequently, the parents/guardians and the patients themselves will be informed by telephone about the project, about their right to withdraw at any time, and the instructions for signing the informed consent. If they are interested to participate, the children and parents are sent the written patient information and informed consent in duplicate, with the request to bring signed copies. If the child is under 16, both parents and guardians must sign the consent form prior to inclusion.

In the plaster room of the Amphia Hospital in Breda, the patient and accompanying parent/guardian receive verbal information about the study again, and the informed consent forms are handed in and signed by the researcher. One copy of the informed consent forms is returned to the patient and parent/guardian.

### Additional consent provisions for collection and use of participant data and biological specimens {26b}

Not applicable.

### Interventions

#### Explanation for the choice of comparators {6b}

In this study, the control group will receive standard care with the addition of a finger pulse oximeter (Onyx Vantage 9590, manufactured by Nonin Medical, Inc., in Plymouth, MN, USA). The intervention group will receive standard care with the addition of a finger pulse oximeter, VR goggles (Oculus Go or Oculus Quest 2, manufactured by Facebook Technologies, LLC in Menlo Park, CA, USA) and headphones (JBL JR300 Junior, manufactured by HARMAN International in Stamford, CT, USA). Standard care consists of the orthopaedic practitioner explaining the procedure and then removing or replacing the plaster. Standard care is chosen as the control, because the purpose is to investigate whether the addition of VR will decrease the anxiety level and thereby improve the patient’s experience during the plaster treatment.

#### Intervention description {11a}

The investigational group will receive standard care and will wear the VR goggles and headphones as well as a finger pulse oximeter during the plaster procedure. The VR goggles will play a video during the procedure to distract the child. The headphones will make sure that the child is more focused on the video by playing the sound of the video and by drowning out the noise of the procedure.

The control group will receive standard care and will wear the finger pulse oximeter.

Each age group (5–11 and 12–17) will have a choice between two videos of approximately 20 min long. The video length is based on the maximum length of the plaster intervention. The children can choose between single episodes of different series on Netflix. The content of the video is based on Netflix recommendations and is screened to make sure the content is appropriate for the hospital setting and the age group.

Children between the ages of 5 and 11 years have a choice between Masha and the Bear season 1 episode 2 and The Thundermans season 1 episode 2; children aged 12–17 years can choose between Modern Family season 1 episode 2 and Brooklyn Nine-Nine season 1 episode 2.

Masha and the Bear is an animated series that focusses on the adventures of a little girl named Masha and a fatherly bear that keeps her safe and prevents disasters. The Thundermans is a comedy series that revolves around the Thundermans, a family with superpowers who try to live normal lives. The spoken language of both videos is set to Dutch.

Modern Family is a sitcom and follows the lives of three diverse families and is filmed from the perspective of an unseen documentary maker. Brooklyn Nine-Nine is a comedy series following the immature but talented NYPD detective Jake Peralta and his diverse, lovable colleagues as they police the NYPD’s 99th precinct. The spoken language of both videos is set to English with Dutch subtitles.

Before the procedure, the child’s anxiety and pain scores are collected with a short questionnaire, using the Child Fear Scale (CFS) [14] and the Numeric Rating Scale (NRS pain), respectively. Then, the orthopaedic practitioner explains the plaster procedure, the finger pulse oximeter is put on and the randomisation result is revealed.

If the child is assigned to the control group, the plaster procedure is then performed by the orthopaedic practitioner. If the child is assigned to the intervention group, the investigator starts the video of choice and places the VR goggles and headphones on the child’s head (adjusting the straps if necessary) before the plaster procedure is performed by the orthopaedic practitioner. During the plaster treatment, the child’s fastest heart rate is recorded by the investigator, using the finger pulse oximeter.

After the procedure, the VR goggles, headphones and finger pulse oximeter are removed. Hereafter, the child’s anxiety and pain scores are collected once more using the CFS^15^ and NRS pain. Additionally, the child and accompanying parent/guardian are asked to rate their satisfaction with the procedure, using the NRS satisfaction.

#### Criteria for discontinuing or modifying allocated interventions {11b}

##### Drop out criteria

Subjects can leave the study at any time for any reason if they wish to do so without any consequences. The parents/guardians of children under the age of 16 can also decide to withdraw their child from the study at any time for any reason without any consequences. The investigator can decide to withdraw a subject from the study for urgent medical reasons.

#### Strategies to improve adherence to interventions {11c}

Not applicable.

#### Relevant concomitant care permitted or prohibited during the trial {11d}

All concomitant care is permitted.

#### Provisions for post-trial care {30}

There will be no separate insurance for subjects participating in this study. The risks associated are not increased, because regular treatment is used in both groups and the VR goggles are just used to distract patients. A request for dispensation from the statutory obligation to provide insurance has been approved. Cover for damage to research subjects through injury or death caused by this study is provided by the liability insurance of the Amphia Hospital.

### Outcomes {12}

The main study outcome parameter is the difference in post-procedural anxiety scores in the intervention and control groups, using the Child Fear Scale (CFS) [15]. The secondary study parameters are the differences between the two groups in anxiety reduction (difference between the child’s CFS score pre- and post-procedural), NRS pain given by the child (pre- and post-procedure), NRS Satisfaction Score given by the child and accompanying parent/guardian and the child’s fastest heart rate measured by a finger pulse oximeter during the procedure.

The following demographic data is collected from the patient’s medical records: date of fracture, date of plaster intervention, age, sex, upper or lower extremity fracture, specific location of the fracture, type of plaster treatment (plaster replacement or removal) and previous experiences with plaster removal. After randomisation, additional demographic data is collected from the patient or accompanying parent/guardian and the orthopaedic practitioner before the plaster intervention (e.g. the use of pain killers, method of plaster removal, and the video chosen (if assigned to the intervention group)).

### Participant timeline {13}

**Fig. 1:**
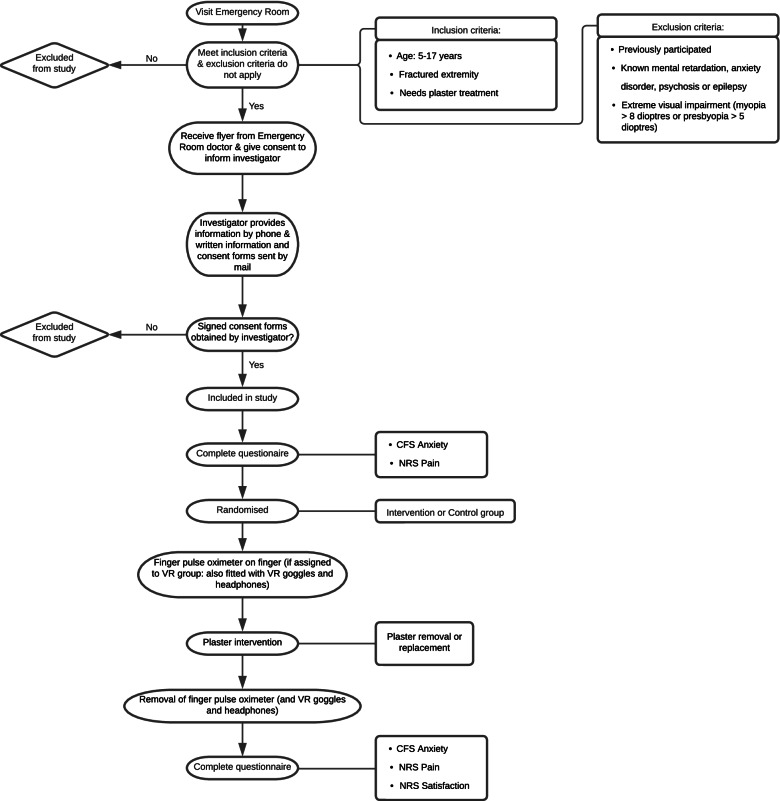
Flowchart of the study

In the emergency room, the patient and parent/guardian are first informed about the study through posters and the informational flyer provided by the attending physician (Fig. 1). The patients and parents/guardians will be further informed about the study by the investigator by phone a few days prior to the scheduled appointment in the plaster room. If they are interested in participating, they will be sent written patient information and consent forms. In the plaster room, the investigator will collect the signed consent forms. The patients are then enrolled in the study and will complete a short questionnaire. Afterwards, the patient will be randomised, and the equipment (finger pulse oximeter, VR goggles and headphones) is placed on the child. The patient will then receive the plaster treatment. Right after the plaster intervention, all the equipment is removed. Lastly, a short questionnaire will be conducted with the child and parent. This concludes the participation of the child in this study.

### Sample size {14}

The expected anxiety score (CFS) in the control group is on average 1.78 ± 1.40 [11]. For an intended improvement by 0.5 (i.e. to 1.28) on the Child Fear Scale, with a statistical power of 80% and an alpha of 0.05, a sample size of 123 participants is needed in each group. To anticipate a 10% dropout, we will include 135 children in each group.

### Recruitment {15}

To help the recruitment and preliminary inform the patients, posters will be put up in the emergency room and the patients will receive a flyer from the physician at the emergency room.

### Assignment of interventions: allocation

#### Sequence generation {16a}

Participants will be randomly assigned to the control or the intervention group with a 1:1 allocation. The randomisation schedule is computer-generated (in Castor Electronic Data Capturing System) and is stratified by age group (ages 5–11 and 12–17) using blocks of random sizes. The block sizes are computer-generated and therefore not known by the investigator. A stratified randomisation is used to ensure that the two groups have a similar distribution in age as this parameter may affect the outcomes [3].

#### Concealment mechanism {16b}

The randomisation is done in Castor EDC. Each patient is randomised after the investigator receives the completed written informed consent forms from the child and/or parent/guardian present and after the first questionnaire is completed. Therefore, the randomisation allocation is not revealed until the patient is included in the study to ensure the allocation group has no effect on the participant’s decision.

#### Implementation {16c}

The allocation sequence will be automatically generated by Castor EDC and is unknown to the investigator. The patients will be enrolled by the investigator, and the randomisation is also initiated by the investigator. The participants will be randomly assigned to the intervention or control group.

### Assignment of interventions: blinding

#### Who will be blinded {17a}

No one is blinded in this study.

#### Procedure for unblinding if needed {17b}

Not applicable.

### Data collection and management

#### Plans for assessment and collection of outcomes {18a}

The following demographics of the patient are collected from the patient’s medical records: date of fracture, date of plaster intervention, age, sex, upper or lower extremity fracture, specific location of the fracture, type of plaster treatment (plaster replacement or removal) and previous experiences with plaster removal. After randomisation, additional demographic data is collected from the patient or accompanying parent/guardian and the orthopaedic practitioner before the plaster intervention (e.g. the use of pain killers, method of plaster removal (if applicable) and the video chosen (if assigned to the intervention group)).

Additionally, data is collected with short questionnaires before and after the procedure. The child’s anxiety and pain scores are collected before and after the procedure, using the Child Fear Scale [15] (CFS) and the Numeric Rating Scale (NRS pain), respectively. The child’s and parent’s satisfaction will be collected after the procedure using the NRS satisfaction.

Lastly, the child’s fastest heart rate (during the procedure) is collected with the finger pulse oximeter.

All data is collected by a single individual. The principal investigator will randomly check the data entry during the study.

#### Plans to promote participant retention and complete follow-up {18b}

Not applicable.

#### Data management {19}

The data is coded by the coordinating investigator of the study. Every patient will be given a consecutive alphanumeric code, consisting of the letters “VR” and three numbers, which will be used for data collection. All data is entered electronically in Castor EDC where all changes to the data will be documented in an audit trail. Only the investigators involved in this study can access and edit the data.

All forms collected for this study, including the signed consent forms, will be stored in locked file cabinets at the research secretariat and will be kept there for 15 years after the completion of the study.

#### Confidentiality {27}

Information about potential participants will be collected through the personal digital patient files (case report forms), which will be used to check the patient’s eligibility to participate in this study and will not be shared. The collected personal information will be digitally stored on a secured drive, only accessible to the investigators involved in this study and by the monitor upon request.

The alphanumeric code assigned to each participant will be used for data collection to maintain confidentiality. The data that is collected in Castor EDC is not retraceable to the patient without the key file, which is securely stored with restricted access.

All forms that contain personal information, such as the signed informed consent forms, will be stored separately from the key file and coded data in a locked file cabinet.

#### Plans for collection, laboratory evaluation and storage of biological specimens for genetic or molecular analysis in this trial/future use {33}

Not applicable.

## Statistical methods

### Statistical methods for primary and secondary outcomes {20a}

The outcomes of both groups will be compared with the use of an independent samples *t*-test or a Mann-Whitney *U* test, depending on the distribution of the data, assessed using a histogram and an independent samples Kolmogorov-Smirnov (K-S test). If the data is normally distributed, an independent *t*-test will be performed, and the mean and standard deviation will be reported. If the data is not normally distributed, a Mann-Whitney *U* test will be performed, and the median and (interquartile) range will be reported.

The statistical package SPSS (version 25, IBM Corp, Armonk, NY, USA) will be used for all statistical analyses. *P*-values < 0.05 are considered significant for the primary outcome. The significance level of the secondary outcomes will be adjusted to multiple testing according to the Holm method [14].

### Interim analyses {21b}

There are no planned interim analyses.

### Methods for additional analyses (e.g. subgroup analyses) {20b}

Descriptive statistics will be provided on the baseline demographic data.

Subgroup analyses will be performed, based on age (5–11 and 12–17), sex (female and male), type of plaster treatment (plaster removal and plaster replacement), method of plaster removal (saw and scissors compared to scissors only), the extremity fractured (upper and lower extremity), the specific location of the fracture (three most frequently found fractures in this study, presumably: distal radius, hand and distal humerus) [16], the use of painkillers and previous experiences with plaster removal.

The statistical package SPSS (version 25, IBM Corp, Armonk, NY, USA) will be used for all statistical analyses.

### Methods in analysis to handle protocol non-adherence and any statistical methods to handle missing data {20c}

We expect the number of withdrawals to be low because the intervention is not invasive and does not increase the risks of the standard procedure. Additionally, all the data will be collected during the visit in the plaster room; there is no follow-up, which will also minimise the missing data. A maximum dropout and/or missing data of 10% is anticipated. We will analyse the data according to the intention-to-treat principle. A complete case analysis will be used.

### Plans to give access to the full protocol, participant-level data and statistical code {31c}

The full anonymised dataset will be available upon request after completion and publication.

### Oversight and monitoring

#### Composition of the coordinating centre and trial steering committee {5d}

Chief investigator (CvB):

- Lead the study design and supervise the conduct of the study
- Advise on selection of patients
- Revise the protocol and amendments
- Ensure Good Clinical Practice
- Agreement of final protocol

Executing Investigator and main author (LvdW):

- The main author of the research proposal and protocol
- Selection and recruitment of patients
- Randomisation and data entry
- Advise on study design
- Train executing investigators on study conduct and data entry
- Obtain informed consent
- Execute data analysis
- Agreement of final protocol

Executing investigator (MP):

- Selection and recruitment of patients
- Randomisation and data entry
- Advise on study design and statistical analyses
- Obtain informed consent
- Agreement of final protocol

Scientist (IvO):

- Revise the protocol and amendments
- Assign a monitor
- Advise on study design and statistical analyses
- Report (serious) adverse events to the METC
- Agreement of final protocol

Advising physician (PvH):

- Revise the protocol and amendments
- Advise on study design
- Agreement of final protocol

#### Composition of the data monitoring committee, its role and reporting structure {21a}

The study will be monitored by a certified individual from the research centre at the beginning (after the first inclusions) and at the end of the study. This person is not involved in this study and is therefore independent from the trial. This monitor will check the patients’ safety and whether the study is executed properly.

#### Adverse event reporting and harms {22}

Adverse events (AEs) are defined as any undesirable experience occurring to a subject during the study procedure, whether or not considered related to VR. All AEs reported spontaneously by the subject or observed by the investigator or his staff will be recorded.

A serious adverse event (SAE) is any untoward medical occurrence or effect that results in death, is life-threatening (at the time of the event), requires hospitalisation or prolongation of existing inpatients’ hospitalisation, results in persistent or significant disability or incapacity, is a congenital anomaly or birth defect or any other important medical event that did not result in any of the outcomes listed above due to medical or surgical intervention but could have been based upon appropriate judgement by the investigator. An elective hospital admission will not be considered as a serious adverse event.

The investigator will report the SAEs through the web portal ToetsingOnline to the accredited METC that approved the protocol, within 7 days of first knowledge for SAEs that result in death or are life-threatening followed by a period of a maximum of 8 days to complete the initial preliminary report. All other SAEs will be reported within a period of maximum of 15 days after the sponsor has first knowledge of the serious adverse events.

#### Frequency and plans for auditing trial conduct {23}

We have no plans to audit the data.

#### Plans for communicating important protocol amendments to relevant parties (e.g. trial participants, ethical committees) {25}

Protocol modifications will be communicated to the relevant parties in the form of an amendment.

#### Dissemination plans {31a}

The results of this study will be presented at scientific meetings and published in a peer-reviewed medical journal.

## Discussion

The aim of this study is to evaluate the effect of distraction by virtual reality on the anxiety levels of children with fractures in the plaster room. Visual distraction with a smartphone or tablet and noise reduction with headphones both have shown a positive effect on the child’s anxiety in different clinical settings [2, 6, 7]. Moreover, VR has successfully reduced both pain perception and anxiety in children in other clinical situations and has proven to be more effective than a tablet [7–12]. Therefore, the combination of visual (with VR goggles) and auditory distraction (with headphones) is hypothesised to provide clear anxiety reduction in the paediatric orthopaedic trauma population described in this protocol.

Recently, Jivraj et al. [13] have studied the effects of VR during plaster removal in a small general paediatric orthopaedic population. They found that the use of VR significantly reduced the child’s anxiety levels. However, there are a few differences in the study design compared with our study. Firstly, the VR set-up differs. Jivraj et al. [13] let the children play a video game with a manual controller, while the present study will show a short video. A video game with a manual controller will not be possible for the majority of our population, i.e. those with upper extremity fractures. Jivraj et al. [13] also mentioned that children under the age of seven found it hard to understand the game and would likely benefit more from a video experience. Secondly, another difference between the two studies is the population type and number. Jivraj et al. [13] included 90 patients with plaster removal for any indication, whereas the present study will include 270 patients in different phases of plaster treatment for fractures only. Due to this difference in population, both studies will provide valuable information on the anxiety-inducing effects of VR in the children treated in plaster room. Thirdly, the instruments to measure anxiety differ between the studies. Where this study uses the CFS and heart rate, Jivraj et al. [13] used the Short State Anxiety Inventory Scale (Short SAIS) for pre- and post-procedural anxiety measurements and the Children’s Emotional Manifestation Scale (CEMS) to measure the anxiety level during the procedure. While both methods are validated methods of measuring anxiety, the CEMS was found to be unpractical in a busy plaster room [13]. However, it will be interesting to study the effects of different methods of anxiety testing on the outcomes in future studies. Lastly, the secondary outcome measures differ between the studies. Jivraj et al. [13] recorded the Penn State Worry Questionnaire for Children (PSWQ-C) scores, nausea level and desire for future use of VR. The secondary outcomes and subanalyses in the present study will therefore add useful information to the knowledge of VR and its effects on outcomes. We believe our study can provide an important contribution to the understanding of children’s anxiety and the role of VR as a distraction method.

## Trial status

This trial commenced on 21 January 2021. The anticipated end date of the study is 1 April 2022

## Data Availability

Only the investigators will have full access to the final dataset.

## Abbreviations

AE: Adverse event
CFS: Child Fear Scale
EDC: Electronic data capture
MEC-U: Medical research Ethics Committees United (one of the METCs)
METC: Medisch Ethische Toetsing Commissie (in English: Medical Ethics Review Committee)
NRS: Numeric Rating Scale
NYPD: New York Police Department
SAE: Serious adverse event
VR: Virtual reality
